# Canning Fruit

**Published:** 1892-09

**Authors:** E. E. Kellogg


### CANNING FRUIT.

Canning consists in sealing in air-tight cans or jars, fruit which has been previously boiled. It is a very simple process, but requires a thorough understanding of the scientific principles involved, and careful management, to make it successful. The result of painstaking effort is so satisfactory, however, it is well worth all the trouble, and fruit canning need not be a difficult matter if attention is given to the following details :

Select self-sealing glass cans of some good variety. Tin cans give more trouble filling and sealing, are liable to affect the flavor of the fruit, and, unless manufactured from the best of material, to impair its wholesomeness. Glass cans may be used more than once and are thus much more economical. Those with glass covers, or porcelain-lined covers are best.

Select only the best of fruit, such as is perfect in flavor and neither green nor overripe. Fruit which has been shipped from a distance, and which is consequently not perfectly fresh, contains germs in active growth, and if the least bit musty, it will be almost sure to spoil, even though the greatest care may be taken in canning.

Cook the fruit slowly in a porcelain-lined or granite-ware kettle, using as little water as possible. It is better to cook only small quantities at a time in one kettle. Steaming in the cans is preferable to stewing, where the fruit is at all soft. To do this, carefully fill the cans with fresh fruit, packing it quite closely, if the fruit is large, and set the cans in a boiler partly filled with cold water, with something underneath them to prevent from breaking — muffin rings, straw, thick cloth, or any thing to keep them from resting on the bottom of the boiler (a rack made by nailing together strips of lath is very convenient); screw the covers on the cans so the water cannot boil into them, but not so tightly as to prevent the escape of steam; heat the water to boiling, and steam the fruit until tender. Peaches, pears, crab apples, etc., to be canned with a syrup, may be advantageously cooked by placing on a napkin in a steamer, over a kettle of boiling water until tender, then dropped into the boiling syrup.

Cooking the sugar with the fruit at the time of canning, is not to be recommended from an economical standpoint; but fruit thus prepared is more likely to keep well than when cooked without sugar; not however, because of the preservative influence of the sugar, which is too small in amount to prevent the action of germs, as in the case of preserves, but because the addition of sugar to the water or fruit juice increases its specific gravity, and thus raises the boiling point. From experiments made, I have found that the temperature of the fruit is ordinarily raised about 5 deg. by the addition of the amount of sugar needed for sweetening subacid fruit. By the aid of this additional degree of heat, the germs are more certainly destroyed, and the sterilization of the fruit will be accomplished in a shorter time.

Use the best sugar, two tablespoonfuls to a quart of fruit is sufficient for most subacid fruits, as berries and peaches; plums, cherries, strawberries, and currants require from five to eight tablespoonfuls of sugar to a quart. Have the sugar hot, by spreading it on tins and heating in the oven, stirring occasionally. See that it does not scorch. Add it when the fruit is boiling. Pears, peaches apples, etc., which contain a much smaller quantity of juice than do berries, may be canned in a syrup prepared by dissolving a cup of sugar in two or three cups of water. Perfect fruit, properly canned, will keep without sugar, and the natural flavor of the fruit is more perfectly retained when the sugar is left out, adding the necessary amount when opened for use.

If the fruit is to be cooked previous to being put in the cans, the cans should be heated before the introduction of the fruit, which should be put in at a boiling temperature. Various methods are employed for this purpose. Some wrap the can in a towel wrung out of hot water, keeping a silver spoon inside while it is being filled; others employ dry heat by keeping the cans in a moderately hot oven while the fruit is cooking.

Another and surer way is to fill a large dish-pan nearly full of scalding (not boiling) water, then gradually introduce each can, previously baked, into the water, dip it full of water, and set it right side up in the pan. When every thing is in readiness, the fruit properly cooked, and at a boiling temperature turn one of the cans down in the water, roll it over once or twice, empty it, and set in a shallow pan of hot water; adjust the funnel, and then place first in the can a quantity of juice, so that when the fruit is put in, no vacant places will be left for air, which is sometimes quite troublesome if this precaution is not taken; then add the fruit. If any bubbles of air chance to be left, work them out with a fork or spoon handle, which first dip in boiling water, and then quickly introduce down the sides of the jar and through the fruit in such a way that not a bubble will remain. Fill the can to overflowing, remembering that any vacuum invites the air to enter; use boiling water or syrup when there is not enough juice. Skim all froth from the fruit, adding more juice if necessary; wipe the juice from the top of the can, adjust the rubber, put on the top, and screw it down as quickly as possible. If the fruit is cooked in the cans, as soon as it is sufficiently heated, fill the can completely full with boiling juice, syrup, or water; run the handle of a silver spoon around the inside of the can, to make sure the juice entirely surrounds every portion of fruit, and that no spaces for air remain, put on the rubbers, wipe off all juice, and seal.—*E. E. Kellogg, in Science in the Kitchen.*

